# Microbiome of Total Versus Live Bacteria in the Gut of Rex Rabbits

**DOI:** 10.3389/fmicb.2018.00733

**Published:** 2018-04-10

**Authors:** Xiangchao Fu, Bo Zeng, Ping Wang, Lihuan Wang, Bin Wen, Ying Li, Hanzhong Liu, Shiqie Bai, Gang Jia

**Affiliations:** ^1^Animal Nutrition Institute, Sichuan Agricultural University, Chengdu, China; ^2^Sichuan Academy of Grassland Science, Chengdu, China; ^3^Farm Animal Genetic Resources Exploration and Innovation Key Laboratory of Sichuan Province, Sichuan Agricultural University, Chengdu, China

**Keywords:** 16S rRNA gene, rabbit, propidium monoazide, microbiota, live bacteria

## Abstract

Gastrointestinal bacteria are essential for host health, and only viable microorganisms contribute to gastrointestinal functions. When evaluating the gut microbiota by next generation sequencing method, dead bacteria, which compose a proportion of gut bacteria, may distort analysis of the live gut microbiota. We collected stomach, jejunum, ileum, cecum, and colon contents from Rex rabbits. A modified propidium monoazide (PMA) treatment protocol was used to exclude DNA from dead bacteria. Analysis of untreated samples yielded total bacteria, and analysis of PMA-treated samples yielded live bacteria. Quantitative polymerase chain reaction and 16S rRNA gene sequencing were performed to evaluate the live-to-total bacteria ratio and compare the difference between live and total microbiota in the entire digestive tract. A low proportion of live bacteria in the foregut (stomach 1.12%, jejunum 1.2%, ileum 2.84%) and a high proportion of live bacteria in the hindgut (cecum 24.66%, colon 19.08%) were observed. A significant difference existed between total and live microbiota. *Clostridiales, Ruminococcaceae*, and *S24-7* dominated the hindgut of both groups, while *Acinetobacter* and *Cupriavidus* dominated only in live foregut microbiota. *Clostridiales* and *Ruminococcaceae* abundance decreased, while *S24-7* increased in live hindgut microbiota. The alpha- and beta-diversities differed significantly between groups. Analysis of networks showed the mutual relationship between live bacteria differed vastly when compared with total bacteria. Our study revealed a large number of dead bacteria existed in the digestive tract of Rex rabbits and distorted the community profile of the live microbiota. Total bacteria is an improper representation of the live gut microbiota, particularly in the foregut.

## Introduction

Gastrointestinal microbiota is essential for host health and nutrient utilization, and only viable microorganisms are capable of contributing to these functions. Until now, only a small number of gastrointestinal microbiota species could be cultured, resulting in a substantial underestimation of their true diversity (Kesmen and Kacmaz, 2011; Pham and Kim, 2012). Although 16S rRNA gene high-throughput sequencing has the ability to more completely evaluate microbiota complexity (Medini et al., 2008), it cannot discriminate between live and dead gut bacteria.

Propidium monoazide (PMA) only penetrates membrane-damaged cells, where a photo-induced azide group covalently binds to DNA. This cross-linking effectively inhibits PCR amplification of DNA from dead cells (Nocker et al., 2006; Bae and Wuertz, 2012) of both Gram-negative and Gram-positive bacteria (Nocker and Camper, 2009). Recently, PMA treatment has been used to study live microbiota in various environments, such as in spacecraft assembly cleanrooms (Vaishampayan et al., 2013), oral cavities (Exterkate et al., 2015), respiratory tracts (Leah et al., 2015), and even activated sludge (Guo and Zhang, 2014). However, high bacterial concentrations (beyond 8 log/ml) and high concentrations of opaque impurities reduce light transparency, thus decreasing the efficacy of PMA-mediated exclusion of dead bacteria (Wagner et al., 2008; Nocker and Camper, 2009; Frankenhuyzen et al., 2011), which, until now, has impeded the application of PMA to gut samples.

Direct 16S rRNA gene sequencing is commonly used to analyze gut microbiota. This can be an error-prone method because of the considerably large percentage of dead bacteria in the gut (Bik et al., 2006; Stearns et al., 2011; Aviles-Jimenez et al., 2014; Bo et al., 2015). Additionally, the variety and distribution of dead microbiota rarely are consistent with that of the live microbiota (Maurice et al., 2013). Furthermore, bacteria which constitute the fecal microbiota are frequently ingested in many species that engage in coprophagy, such as lagomorphs, rodents, foals, dogs, and non-human primates (Soave and Brand, 1991). Such exogenous bacteria may be killed in stomach and seriously interfere with the composition of live microbiota in the foregut.

Hence, given Rex rabbits frequently consuming soft feces (Hörnicke et al., 1984; Emaldi et al., 2008), we selected them as a representative species and hypothesized that: (1) The dead bacteria may significantly interfere with analyzing the composition of live bacteria in the whole digestive tract; (2) This interference may be particularly serious in the foregut due to coprophagy in rabbits. In this study, live bacteria were separated from dead bacteria based on modified PMA treatment, and then the difference between total and live microbiota was compared.

## Materials and Methods

### Experimental Design and Sampling

**FIGURE 1:**
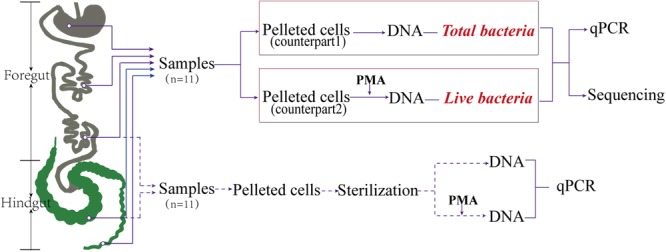
Sites sampled and key steps. The five sites sampled in the gut were categorized as foregut (stomach, jejunum, ileum) and hindgut (cecum and colon). The red italicized “*Total bacteria*” represents the non-PMA treatment group. The red italicized “*Live bacteria*” represents the PMA treatment group.

All animal experiments were approved by the Institutional Animal Care and Use Committee of the Sichuan Agricultural University (#DKY-B20141509) and were performed at the Rex rabbit breeding center in accordance with the relevant regulations. All Rex rabbits received customized fodder without probiotics or antibiotics (Supplementary Table S1). Briefly, 120 rabbits (six rabbits per litter, 20 L) born on the same day were raised in separate cages after weaning (day 40). Rabbits were permitted to feed and drink *ad libitum*. At day 90, 11 female rabbits with similar weight (from 1,846 to 1,921 g, average = 1,869 g) were selected from different litters to minimize environmental and genetic variation and were sacrificed after 12 h of fasting. Intestinal contents from the foregut (stomach, jejunum, and ileum) and the hindgut (cecum and colon) were collected (**Figure 1**), and the samples were mixed with glycerol to reach a final concentration of 15% to facilitate the storage of live bacteria (Hollander and Nell, 1954; Crowther, 1971), frozen in liquid nitrogen, then stored at -80°C.

### Pretreatment of Samples

Impurities were removed using the method previously described (Guo and Zhang, 2014; Heise et al., 2015). Approximately 0.2 g of frozen samples were transferred to 2-ml microcentrifuge tubes with 1.8 ml 1× phosphate-buffered saline (PBS), incubated for 2 min, vortexed for 2 min, and centrifuged for 4 min at 600 × *g*. The liquid was transferred to another 2-ml tubes using wide-mouthed pipette tips. The above steps were repeated with the residuals to improve the elution ratio. The liquid was then centrifuged for 8 min at 5000 × *g*, and the supernatant was discarded. For PMA efficiency validation, the samples obtained from the ileum and cecum were sterilized at 95°C for 30 min. The pellets were re-suspended in 500 μL of 1× PBS for subsequent PMA treatment. Subsequently, the optical density at 600 nm (OD_600_) of the samples was measured using a TU-1810 ultraviolet and visible spectrophotometer (Persee, Beijing, China). In order to understand the effect of modified PMA treatment protocol on live bacteria, paralleled samples were prepared and eluted, bacterial cells in supernatants were collected (12,000 × *g*) and used to validate the ratio of lost live bacteria. Pelleted cells experienced the process once again to validate the ratio of lysed live bacteria.

### PMA Treatment

To compare differences between total and live microbiota in the entire gut, samples were divided into two groups after pretreatment, with each group containing 55 samples from the same areas of the gut. Group 1, which was not treated with PMA, represented total bacteria and was referred to as the control group; Group 2, which was treated with PMA, represented live bacteria (**Figure 1**).

The PMA treatment protocol was modified by adding a thin-layer step to increase light transmission. The PMA concentration, incubation time, and illumination conditions previously described were followed (Bae and Wuertz, 2009; Heise et al., 2016). Briefly, PMA (Biotium, Inc., Hayward, CA, United States) was dissolved in 20% dimethyl sulfoxide (DMSO) to create a storage liquid of concentration of 20 mM, which was stored at -20°C in the dark. Storage liquid (2.5 μL) was added to adjust the final concentration of PMA to 100 μmol/L. Samples were incubated for 5 min in the dark at room temperature with occasional inversion, then transferred into transparent bags (DNA-free, 7 cm × 10 cm) (Xinlin Plastic, Chengdu, China), and subsequently flattened to an approximately 0.1 mm thick layer at the bottom of the bag (∼35 cm^2^ area). The sample bags were laid out horizontally on ice 20 cm below the light source. For PMA activation, the samples were exposed to light for 8 min using a 650 W halogen light source. The samples were then transferred to 2-ml microcentrifuge tubes. The bacterial cells were pelleted by centrifugation at 5,000 × *g* for 8 min and stored at -20°C.

### DNA Extraction and Quantitative Polymerase Chain Reaction

Total bacterial DNA was extracted with the TIANamp Stool DNA Kit (TIANGEN Biotech, Beijing, China). The extraction was conducted according to the manufacturer’s instructions, and the DNA was stored at -80°C until further use.

Quantitative polymerase chain reaction (qPCR) was performed on a qTOWER 2.2 (Analytik Jena AG, Germany), using Extaq premix (Takara, Dalian, China). DNA was amplified with the 331F/797R (Nadkarni et al., 2002) primer set (331F: TCC TAC GGG AGG CAG CAG T, 797R: GGA CTA CCA GGG TAT CTA ATC CTG TT) and the same reaction conditions previously reported were used (Wang et al., 2012).

### 16S rRNA Gene Sequencing and Analysis

16S rRNA gene sequencing was performed by the Novogene Bioinformatics Technology, Co., Ltd. Briefly, DNA was amplified using the 341F/806R (Berg et al., 2012) primer set (341F: CCT AYG GGR BGC ASC AG, 806R: GGA CTA CNN GGG TAT CTA AT) with the barcode, targeting the V3+V4 region of the bacterial 16S rRNA gene. The PCR was carried out with Phusion^®^ High-Fidelity PCR Master Mix (New England Biolabs) with the following cycle conditions: 98°C for 30 s, followed by 30 cycles of 98°C for 10 s, 60°C for 20 s and 72°C for 20 s and a final extension of 72°C for 10 min. After PCR amplification, and gel electrophoresis checking, PCR products was purified with Qiagen Gel Extraction Kit (Qiagen, Germany). Sequencing library were generated using TruSeq^®^ DNA PCR-Free Sample Preparation Kit (Illumina, United States). Sequencing was performed using Illumina MiSeq 2 × 250 platform.

Paired-end reads were assigned to samples based on their unique barcodes and primer sequence and merged using FLASH (Mago and Salzberg, 2011). Quality filtering were performed under specific filtering conditions to obtain the high-quality clean tags (Bokulich et al., 2013) according to the QIIME quality controlled process (Caporaso et al., 2010). Chimeras were removed using USEARCH8.0 (Edgar and Flyvbjerg, 2015). The sequencing data were deposited in the NCBI Sequence Read Archive (Accession No. SRP108996). Clean data were analyzed via QIIME (v1.9.1), using Python scripts (Caporaso et al., 2010). Operational taxonomic units (OTUs) were picked using the *de novo* OTU picking protocol with a 97% similarity threshold. Taxonomy assignment of OTUs was performed by comparing sequences to the Greengenes database (gg_13_5_otus). The dominant bacteria were visualized using iTOL (Ganju et al., 2016). Alpha diversity as indicated by Chao1 and the Shannon index was calculated. Beta diversity was calculated on the basis of the weighted UniFrac distance (Lozupone and Knight, 2006). Enterotype analysis was performed by using R (Ade4 package) (Thioulouse and Dray, 2007) based on the Jensen–Shannon distance, partitioning around medoid clustering (PAM), and Calinski-Harabasz Index (CH) at the genus level (Arumugam et al., 2011), and visualized via principal coordinate analysis (PCoA).

Network speculation was performed via CoNet (Faust et al., 2012). Co-occurrence networks were constituted based on the union of subnets (stomach-jejunum, jejunum-ileum, ileum-cecum, and cecum-colon) to display the relationships of gut microbiota within sites and between adjacent sites. The subnets were determined according to the relationship between bacterial abundance; Spearman’s rank correlation coefficient, mutual information, Bray–Curtis dissimilarity, and Kullback-Leibler divergence were calculated; an automatic threshold (positive and negative 3000 edges) was chosen in the stomach-jejunum subnet of total bacteria, and applied to other subnets. One thousand permutations and bootstrap scores were generated. Edges with merged *P*-values < 0.01 were kept (Benjamini–Hochberg correction). Networks were visualized via Cytoscape 3.4 (Killcoyne et al., 2009), applying the Networkanalyzer plugin analysis for network characteristics.

The Mann–Whitney *U*-test was used to examine the statistical significance of the alpha and beta diversities and the degree of networks via SPSS v22.0. Analyses of similarities (ANOSIM) was used to test the similarity between sites or groups via scripts of QIIME.

## Results

### Efficacy of PMA Treatment

**FIGURE 2:**
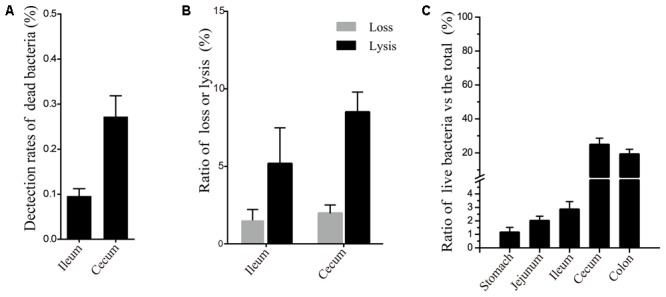
Efficacy of PMA treatment and the ratio of live bacteria in gut samples. **(A)** PMA-treatment efficacy in heat-sterilized pelleted cells from the ileum and cecum. The length of the bar corresponds to the ratio of residual dead bacteria. **(B)** Ratio of loss and lysis of live bacteria during PMA treatment. “Loss” represents the ratio of lost live bacteria along with supernatants during elution. “Lysis” represents the ratio of lysed live bacteria in the whole process. **(C)** Ratio of live bacteria in different sites sampled. The length of the bar corresponds to the ratio of live bacteria.

We arranged heat-sterilized samples to validate the efficacy of PMA as compared to previous reports (Nocker et al., 2006). Ileal samples representing those of the foregut and cecal samples representing those of the hindgut (**Figure 1**) transmitted light similarly as evaluated by OD_600_ (Supplementary Figure S1). PMA treatment induced a significant decrease in dead bacteria as compared with that in non-PMA-treated samples. The percentage of residual dead bacteria was 0.097% in the ileum and 0.27% in the cecum (**Figure 2A**). PMA treatment effectively prevented the dead bacteria DNA from being detected in foregut or hindgut. Therefore, the entire treatment process resulted in very low loss of live bacteria (<2%) and low percentages of bacterial lysis (5.1 ± 1.5% ileum, 8.4 ± 1.7% cecum) (**Figure 2B**).

### Ratio of Live Bacteria in Gut Samples

Quantitative PCR revealed low percentages of live bacteria in the foregut (stomach 1.12%, jejunum 1.2%, and ileum 2.84%). It also showed high percentages of live bacteria in the hindgut (cecum 24.66% and colon 19.08%) (**Figure 2C**).

### Samples and 16S rRNA Gene Sequencing

After quality control review of the samples in both groups, 102 digestive samples (50 control and 52 PMA-treated) were sequenced. For data analysis, chimera checking and singleton OTU filtering were performed, resulting in a total of 3,902,074 high-quality sequence reads, assigned to 45,813 OTUs. On average, each sample contained 3,292 OTUs and 38,255 reads (Supplementary Table S2).

### Changes in Bacterial Composition

**FIGURE 3:**
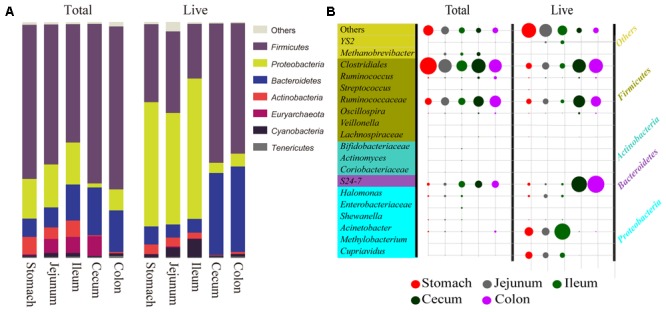
Comparison of predominant bacteria between total and live microbiota at two taxonomic levels. **(A)** Comparison of seven dominant bacteria at the phyla level in the two groups. The length of the bar corresponds to the relative bacterial abundance. **(B)** Comparison of 20 dominant bacteria at the genus level in the two groups. The size of the circle corresponds to relative bacterial abundance. “Total” represents total bacteria, the control; and “Live” represents live bacteria remaining after PMA treatment.

The main discrepancy between total and live microbiota consisted of changes in the abundance of dominant bacteria (**Figure 3A**). The most dominant phylum for total bacteria in the entire gastrointestinal tract was Firmicutes. The second most dominant in the foregut was Proteobacteria and in the hindgut was Bacteroidetes. Comparing the profiles of live and total bacteria, a change in the ranking of most abundant phyla in the foregut was observed. But in the hindgut these changes were less severe, as the relative abundance of Firmicutes and Bacteroidetes changed only slightly. These findings demonstrate that dead bacteria are a more substantial interference to the foregut microbiota.

At the genus level (**Figure 3B**), Acinetobacter was the most dominant among live groups and was dramatically increased in foregut when compared to total groups. *Cupriavidus* was also abundant, suggesting that dead bacteria severely interfered with live bacteria in the foregut as well. The abundance of *Clostridiales* decreased throughout the entire digestive tract in the live groups, but this decrease was more evident in the foregut. *Ruminococcaceae* showed a slight decrease and *S24-7* exhibited an increase in the hindgut, suggesting that the distortion of dead to live bacteria was still severe for certain bacteria in the hindgut at the genus level.

### Changes in Alpha-Diversity

**FIGURE 4:**
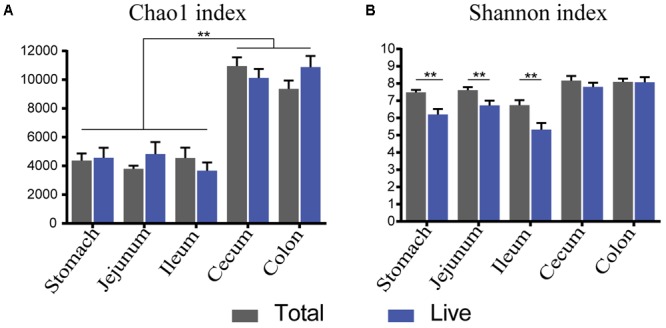
α-Diversity trends between total and live microbiota. Bar charts illustrate a comparison of diversity indices (**A**: Chao 1, **B**: Shannon index). ^∗^*P* < 0.05, ^∗∗^*P* < 0.01.

Both total and live groups exhibited a reduced Chao1 index in the foregut compared to the hindgut (**Figure 4A**, *P* < 0.01), and yet Chao1 index had not significant difference between paired sites for both groups (**Figure 4A**, *P* > 0.05). The Shannon diversity index of live bacteria was significantly lower than that of total bacteria in the foregut (**Figure 4B**, *P* < 0.05). Total bacteria had the same alpha-diversity as live bacteria in the hindgut (**Figure 4**, *P* > 0.05). Interference from dead bacteria lead to an increase of Shannon diversity index in the foregut but not in the hindgut.

### Beta-Diversity of Total and Live Microbiota

**FIGURE 5:**
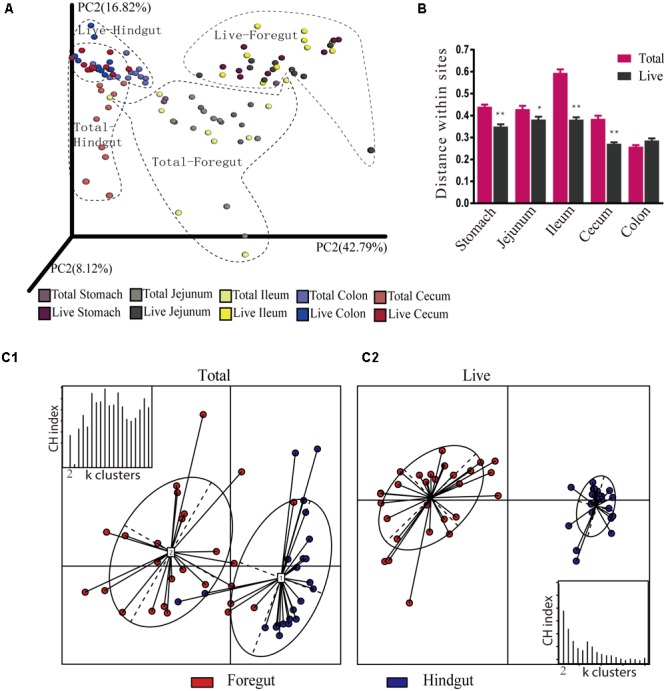
Jackknifed β-diversity between total and live microbiota at OTU and genus levels. **(A)** Principal coordinate analysis (PCoA) of control and PMA-treated microbiota based on weighted UniFrac distances at the OTU level. Each point on the PCoA plots represents the average related location of the microbiota of each sample. **(B)** UniFrac distance within sites of total and live bacteria. **(C)** PCoA of control and PMA-treated microbiota based on JSD distance at the genus level. The nodes that exhibit an irregular ellipse cover are corresponding to the substructure of the gastrointestinal tract. CH index: Calinski-Harabasz Index; k clusters: partitioning around medoids (PAMs) clustering algorithm to cluster abundance profiles. ^∗^*P* < 0.05, ^∗∗^*P* < 0.01.

A comparison between the total bacteria and live bacteria groups demonstrated significant differences in bacterial distribution between paired sites (**Figure 5A**, ANOSIM: *R* = 0.193, *P* < 0.001). A comparison of the microbiota within the respective groups demonstrated significant similarities between sites within the foregut (**Figure 5A**, ANOSIM: total bacteria *R* = 0.071; Live bacteria *R* = 0.086, *P* > 0.05), while a significant difference between sites in the hindgut was observed (**Figure 5A**, ANOSIM: *R* = 0.393, *P* < 0.001). Furthermore, within each group, the microbiota was significantly different between the foregut and the hindgut (**Figure 5A**, ANOSIM: total bacteria *R* = 0.629; Live bacteria *R* = 0.656, *P* < 0.001), suggesting that microbiota of the entire digestive tract could be divided into two clusters. A comparison of the diversity within sites showed that the diversity of total bacteria was greater than that of live bacteria in corresponding sites (**Figure 5B**, Mann–Whitney *P <* 0.05), with the exception of the colon. This indicates that dead bacteria caused significant differences in diversity mainly in the foregut and cecum.

In order to confirm whether physiological sites possessed their own microbiota, enterotype analysis was performed for the respective groups at the genus level (**Figure 5C**). For total bacteria, the optimal K value was 10, which exceeded the five sites sampled. We arbitrarily chose 2 as the optimal K value to match the sections examined (foregut and hindgut). The distance between both enterotypes was closer, and the distribution of samples was more discrete when four samples from the foregut were incorrectly clustered into the hindgut (**Figure 5C1**). For live bacteria, samples were optimally clustered into two enterotypes, but the microbiota of the foregut and hindgut, respectively, clustered more closely together (**Figure 5C2**). This suggests that dead bacteria severely interfered with the composition of live bacteria in foregut and hindgut.

### Changes in Co-occurrence Networks

**FIGURE 6:**
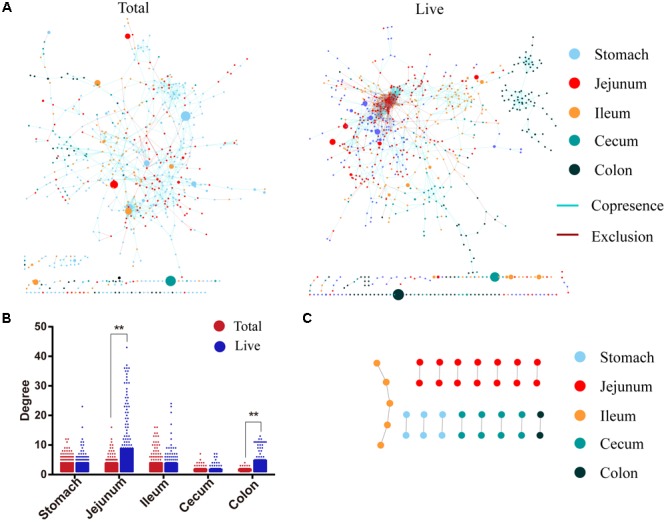
Different co-occurrence network. **(A)** Co-occurrence network of total and live microbiota. The connections represent a stronger correlation (Pearson: –0.52, Spearman: –0.62, Mutual information: 0.60, Bray–Curtis dissimilarity: 0.25, Kullback-Leibler dissimilarity: 0.49). Nodes represent taxa in the data sets. The size of each node is proportional to the relative abundance. **(B)** Node degree values associated with different digestive sites. **(C)** Intersection of both co-occurrence networks. ^∗∗^*P* < 0.01.

We constructed two co-occurrence networks (**Figure 6A**) based on the union of four subnets for the respective groups. Primary network characteristics showed fewer differences for total bacteria vs. live bacteria: the large number of nodes and edges were captured (**Figure 6A**, 735/1933 vs. 627/1145); networks followed a power-law distribution (**Figure 6A**, 0.97, R2 0.86 vs. 0.89, R2 0.88); and the average degree in the hindgut was higher than in the foregut (**Figure 6A**, 4.05/1.96 vs. 4.67/2.67).

However, the two networks showed large differences in other characteristics. The degrees of total bacteria in the jejunum and colon were lower than that of live bacteria (jejunum 2.98 vs. 7.55, colon 1.47 vs. 3.50, Mann–Whitney *P* < 0.01) (**Figure 6B**). The network contained only 8% negative correlation for total bacteria, while negative correlations as high as 30% were observed for live bacteria, and 90.5% for live bacteria in the foregut. When the two networks formed an intersection, only 35 nodes and 19 edges could be found (**Figure 6C**), indicating that both networks were almost completely different.

## Discussion

The efficacy of the modified PMA treatment protocol was confirmed in the sterilized samples. The dead bacteria were almost entirely removed; less than 0.27% dead bacteria escaped PMA treatment, approaching the efficacy of traditional PMA treatment for samples with low turbidity (Nocker et al., 2007; Bae and Wuertz, 2009; Yáñez et al., 2011). Furthermore, the entire treatment process resulted in very low loss and lysis of live bacteria. We could not completely avoid the loss of live bacteria because sample pretreatment inevitably included harmful processes, such as exposure to oxygen and high pressures (Munukka et al., 2012). Therefore, we accepted the surviving microbiota after PMA treatment as live bacteria.

The percentage of live bacteria was low throughout the entire digestive tract. However, samples collected from the hindgut had the higher ratio of live bacteria, which is approximate with prior results obtained by culture (Emaldi et al., 2008). This may explain the role of the cecum in providing the body with volatile fatty acids (VFAs), mycoprotein, and vitamins (Gidenne, 1997) and the large number of live bacteria needed to realize these functions. The foregut had a smaller ratio of live to total bacteria in sampled sites, this may have been the result of gastric acid killing the soft feces bacteria ingested. Whereas the dead bacteria may have significantly interfered with the composition of live bacteria in hindgut like reports in human (Benamor et al., 2005; Maurice et al., 2013), more seriously in foregut due to supplementary soft feces bacteria.

Comparison of the total versus live microbiota validated our speculations. A significant difference was found in the bacterial composition of the total microbiota when compared with that of the live microbiota in the entire digestive tract. Firmicutes was the most dominant phylum among total bacteria in the foregut; however, the most dominant bacterial phylum in live microbiota was Proteobacteria. Coprophagic mice have shown similar microbiota composition (Gu et al., 2013). In chickens, though not coprophagic, the microbiota of the foregut was also similar to that of the craw (Choi et al., 2014), and Firmicutes was the most prevalent bacterial phylum in the foregut and hindgut of both animals. Moreover, species that do not ingest exogenous bacteria, such as humans (Stearns et al., 2011) and swines (Zhao et al., 2015), generally displayed greater discrepancies between foregut and hindgut, with Proteobacteria predominant in the foregut. Obviously, exogenous bacteria exacerbate the more serious interference with live microbiota in the foregut.

Analysis of beta-diversity further demonstrated significant differences between the total versus live micobiota. Shannon diversity was significantly reduced in live microbiota. Community richness did not significantly change after ingesting exogenous bacteria from soft feces; perhaps the core microbiome was the same in the entire gastrointestinal tract of the Rex rabbits as it is in cattle (Mao et al., 2015) or mice (Gu et al., 2013).

Although we observed significant differences between total and live microbiota, dead bacteria only caused moderate disturbance to live microbiota in the hindgut. The rabbit soft feces may supplement less exogenous dead bacteria into the hindgut microbiota. Soft feces only account for about 1/5 of gross feces (Gidenne and Lapanouse, 2010) and associated bacteria are digested in foregut. Endogenous dead bacteria were the main source interfering with live hindgut microbiota, like other non-coprophagic species (Maurice et al., 2013). The order of dominant bacteria was not reversed like that in the foregut and the presence of dead bacteria did not give rise to significant differences in richness and Shannon diversity.

We also observed significant diversity variation within sites in total bacteria, which means large individual differences, particularly in the foregut and cecum. Previous studies have reported broad differences of microbiota in many species as well (Turnbaugh et al., 2009; Cantarel et al., 2011). The individual difference was significantly reduced in live bacteria of Rex rabbits, which means that live microbiota has less errors. Analysis of enterotypes further validated this part. The distinctive microbiota belonging to foregut or hindgut can be accurately clustered into two; a parallel phenomenon has been described in cattle (Mao et al., 2015). Perhaps, as a rule of host-to-gut microbiota (Delong, 2014), specific physiological sites of the gut corresponded with specific microbiota in rabbits. Yet, the presence of the dead bacteria confounded the analysis and division of the enterotypes. And then, a question raised is whether other species may have similarly reduced diversity of live bacteria. The reduced intraspecies variation seen in the analysis of live bacteria versus total bacteria translates to “less noise,” which creates a more favorable environment to reveal differences that would otherwise be obscured by dead bacteria.

Network analysis was generally used to deduce microbiome with commensal, mutual, parasitic, competitive, and partial-obligate-syntrophic ecological models (Faust et al., 2012; Ma et al., 2016; Weiss et al., 2016). In this study, the basic characteristics of the network were similar for total and live microbiota. Yet, both co-occurrence networks were almost completely different due to very small intersections. Live bacteria had a large number of negative correlations in the foregut, which is a negative feedback system, and could explain the stability of the foregut microbiota (Coyte et al., 2015). Yet, dead bacteria obscures the evaluation of these relationships between live bacteria.

Mice as a model animal were frequently applied to gastrointestinal microbiota research (Carroll et al., 2012; Scaldaferri et al., 2014). Given this serious distortion of foregut microbiota in coprophagic animals, the use of mice to study human conditions thought to be attributable to pathogenic bacteria in the small intestine, such as inflammatory bowel disease, which is commonly investigated by direct 16S rRNA gene sequencing (Lyra et al., 2009), could result in unreliable results.

In summary, a high percentage of dead bacteria existed in the entire digestive tract, which interfered with live bacteria. Thus, total bacteria was an inaccurate representation of live bacteria and has less biological significance in comparison with live bacteria. Ingestion of soft feces appeared to cause more extensive interference with foregut microbiota. Therefore, future studies evaluating gastrointestinal microbiota should focus on live bacteria, particularly studies using models of coprophagic animals, such as mice or rabbits.

## Ethics Statement

All applicable international, national, and institutional guidelines for the care and use of animals were followed.

## Author Contributions

GJ, XF, and BZ: conceived and designed the experiments. XF, PW, LW, BW, SB, HL, YL, and GJ: performed the experiments. XF, YL, BZ, and GJ: wrote the paper.

## Conflict of Interest Statement

The authors declare that the research was conducted in the absence of any commercial or financial relationships that could be construed as a potential conflict of interest.
