# Supplementary material for: Microbiome of Total Versus Live Bacteria in the Gut of Rex Rabbits
Source: Front Microbiol. 2018 Apr 10;9:733. doi: 10.3389/fmicb.2018.00733 (PMC5902543; doi:10.3389/fmicb.2018.00733)
Supplement: Supplementary file 1 [file Data_Sheet_1.PDF]

Microbiome of Total versus Live Bacteria in the Gut of Rex Rabbits

Xiangchao Fu[1,2]†, Bo Zeng[3]†, Ping Wang[2], Lihuan Wang[2], Bin Wen[2], Ying Li[3], Hanzhong Liu[2], Shiqie Bai[2*] and Gang Jia[1*]

[1] Animal Nutrition Institute, Sichuan Agricultural University, Chengdu, Sichuan, China.

[2] Sichuan Academy of Grassland Science, Chengdu, Sichuan, China.

[3] Farm Animal Genetic Resources Exploration and Innovation Key Laboratory of Sichuan Province, Sichuan Agricultural University, Chengdu, Sichuan, China.

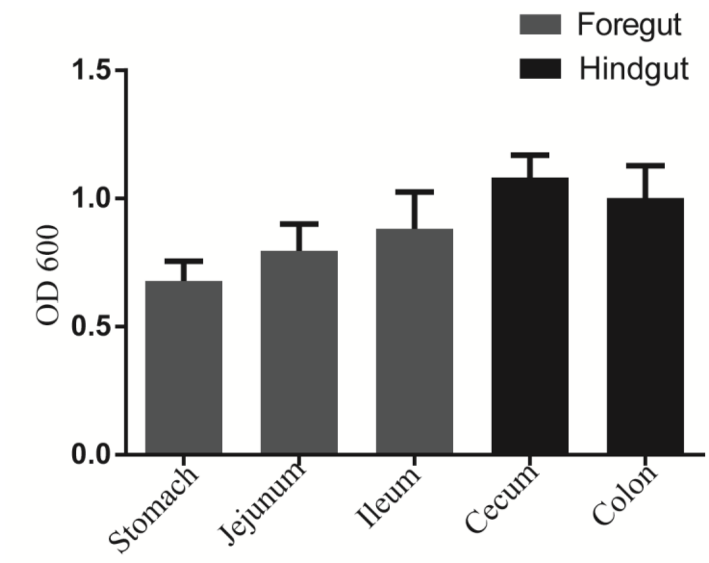

Figure S1: Comparison of light-transmission of samples on basis of OD600. OD600 of samples eluted were measured for diluents in 100 times on basis of change of sample thickness before and after thin-layer treatment.

Diluents in 100 times was on basis of the following calculation: light-transmission distance for samples in microcentrifuge tubes was equal to diameter of tubes (1.4 cm). When samples (0.5 ml about 0.5 cm3) were flatten into plastic bags (7 cm×10 cm), light-transmission distance was equal to thickness of samples in bags (0.5 cm^3/ (7 cm×10 cm) = 0.014 cm). Finally, light-transmission distance reduced about 100 times before and after thin-layer treatment.

Table S1 Composition and nutritional ingredients of diet

Composition	Percentage	Ingredient	Content
Maize (%)	20	Digestible Energy（MJ/Kg）[2]	10.37
Wheat bran (%)	10	Dry Matter (%)	88.2
Soybean meal (%)	17	Crude Protein (%)	17.21
Alfalfa (%)	35	Starch (%)	14.96
Rice chaff (%)	16	Crude Fiber (%)	17.23
Premix[1] (%)	2	Neutral Detergent Fiber (%)	35.07
		Acid Detergent Fiber (%)	22.33
		Water Soluble Cellulose (%)	17.01

1）The premix provides following per kg diet：Fe(as ferrous sulfate) 40 mg，Cu (as copper sulfate) 30 mg，Zn (as zinc sulfate) 20 mg，Mn10 mg，Mg 20mg，Nacl 400mg，VA 900 IU，VD3 600 IU，VE 60 IU。

2）DE is calculated value. Other nutrient levels are measured values.

Table S2 Sample metadata and OTU table summary

Sample metadata				
**#Sample_ID**	**#Treatment**	**#Segment**	**#OTUs**	**#Sequence_counts**
B1M	Control	Cecum	4603	40757
B2M	Control	Cecum	3586	36120
B3M	Control	Cecum	5251	37809
B4M	Control	Cecum	5569	36844
B5M	Control	Cecum	5526	46420
B6M	Control	Cecum	5035	36062
B7M	Control	Cecum	5110	42958
B8M	Control	Cecum	5026	41757
B9M	Control	Cecum	4664	39742
B10M	Control	Cecum	4569	37669
B11M	Control	Cecum	2717	32935
B1H	Control	Ileum	3885	45563
B2H-B3H	Control	Ileum	2347	38202
B3H	Control	Ileum	3358	47747
B4H	Control	Ileum	2003	48499
B5H	Control	Ileum	2110	44393
B7H	Control	Ileum	1091	34116
B8H	Control	Ileum	1385	36262
B9H	Control	Ileum	1230	39637
B10H	Control	Ileum	1210	43300
B11H	Control	Ileum	1201	40640
B1K	Control	Jejunum	3168	38608
B2K	Control	Jejunum	4776	40708
B3K	Control	Jejunum	3961	42785
B4K	Control	Jejunum	3196	39118
B5K	Control	Jejunum	2669	46575
B6K	Control	Jejunum	1654	36044
B7K	Control	Jejunum	1746	37000
B8K	Control	Jejunum	1996	44169
B9K	Control	Jejunum	1162	40491
B10K	Control	Jejunum	1370	35931
B11K	Control	Jejunum	1378	33376
B1J	Control	Rectum	4794	37408
B2J	Control	Rectum	4554	39038

B3J	Control	Rectum	5502	40398
B4J	Control	Rectum	5534	39810
B5J	Control	Rectum	5701	41229
B6J	Control	Rectum	5136	37327
B7J	Control	Rectum	5605	36620
B8J	Control	Rectum	6404	42817
B9J	Control	Rectum	6120	45364
B10J	Control	Rectum	4980	40303
B11J	Control	Rectum	2110	36006
B1W	Control	Stomach	2558	38598
B2W	Control	Stomach	2749	36259
B3W	Control	Stomach	2915	36892
B4W	Control	Stomach	2578	37524
B5W	Control	Stomach	3075	42906
B7W	Control	Stomach	1252	32604
B8W	Control	Stomach	1235	37625
B9W	Control	Stomach	1460	38816
B11W	Control	Stomach	997	39656
B1MP	PMA	Cecum	4699	36130
B2MP	PMA	Cecum	4495	31182
B3MP	PMA	Cecum	4685	27494
B5MP	PMA	Cecum	5705	38187
B6MP	PMA	Cecum	4356	26370
B7MP	PMA	Cecum	5128	39260
B8MP	PMA	Cecum	5665	32560
B9MP	PMA	Cecum	5579	33630
B10MP	PMA	Cecum	4640	47062
B11MP	PMA	Cecum	3242	43803
B1HP	PMA	Ileum	2443	27451
B2HP	PMA	Ileum	2108	35304
B4HP	PMA	Ileum	1838	31397
B5HP	PMA	Ileum	4151	40731
B6HP	PMA	Ileum	1981	40753
B7HP	PMA	Ileum	1423	23440
B8HP	PMA	Ileum	2461	35246
B9HP	PMA	Ileum	2457	38747
B10HP	PMA	Ileum	1748	40100
B11HP	PMA	Ileum	2196	39683
B1KP	PMA	Jejunum	1807	23051
B2KP	PMA	Jejunum	2530	24939
B3KP	PMA	Jejunum	2100	33467

B5KP	PMA	Jejunum	2241	33684
B6KP	PMA	Jejunum	1911	34404
B7KP	PMA	Jejunum	1697	36732
B9KP	PMA	Jejunum	1604	39761
B10KP	PMA	Jejunum	2401	31368
B11KP	PMA	Jejunum	2614	36200
B1JP	PMA	Rectum	4788	32209
B2JP	PMA	Rectum	3877	44733
B3JP	PMA	Rectum	4730	48412
B4JP	PMA	Rectum	4302	39860
B5JP	PMA	Rectum	5245	41433
B6JP	PMA	Rectum	4475	42971
B7JP	PMA	Rectum	4841	41561
B8JP	PMA	Rectum	4161	45010
B9JP	PMA	Rectum	5149	49552
B10JP	PMA	Rectum	3614	44214
B11JP	PMA	Rectum	2922	40179
B1WP	PMA	Stomach	3317	43907
B2WP	PMA	Stomach	3457	31545
B3WP	PMA	Stomach	3201	49761
B4WP	PMA	Stomach	2390	28797
B5WP	PMA	Stomach	1944	25602
B6WP	PMA	Stomach	2166	46430
B7WP	PMA	Stomach	1878	30830
B8WP	PMA	Stomach	1829	35148
B9WP	PMA	Stomach	1853	33539
B11WP	PMA	Stomach	1965	44808

**OTU table summary**				
Num samples: 102				
Num observations: 45813				
Total count: 3902074				
**Counts/sample summary**			**Observations/ sample summary**	

Min	23051		Min	997
Max	49761		Max	6404
Median	38677.5		Median	2998.5
Mean	38255.627		Mean	3292.353
Std. dev.	5721.678		Std. dev.	1509.09
